# Information, Sharing, and Self-Determination: Understanding the Current Challenges for the Improvement of Pediatric Care Pathways

**DOI:** 10.3389/fped.2020.00371

**Published:** 2020-07-08

**Authors:** Matteo Scopetti, Alessandro Santurro, Vittorio Gatto, Martina Padovano, Federico Manetti, Stefano D'Errico, Vittorio Fineschi

**Affiliations:** ^1^Department of Anatomical, Histological, Forensic and Orthopedic Sciences, Sapienza University of Rome, Rome, Italy; ^2^Department of Medicine, Surgery and Health Sciences, University of Trieste, Trieste, Italy

**Keywords:** pediatrics, ethics, shared decision-making, care planning, end of life, healthcare quality

## Abstract

Despite the considerable progress of medical science over the years, pediatric patients can still be affected by serious illnesses that, regardless of age, lead to experiencing all the clinical, psychological, ethical and spiritual problems related to incurable diseases and death. The interaction between the peculiarities of individuals, and the clinical conditions presented define a changing and complex profile of health needs, which requires organized, dynamic and multidimensional responses. The approach to the pediatric patient must consider its biological, psychological, relational and clinical characteristics. Such aspects in fact determine and modulate the type and quantity of the needs presented, conditioning the actions to be taken and the organizational models to be implemented. In accordance with some international regulations, it is essential that healthcare professionals provide adequate information to the patient's understanding in order to enhance participation in the decision-making process regardless of the possibility of expressing consent or dissent to the treatment. Frequently, the sharing of decisions on the care path not only fails to involve children, but often lacks rigorously designed interventions for parental involvement. Therefore, the development of care models that focus on the needs of the pediatric population is crucial. The present paper aims to analyze the problems of information quality and sharing in pediatric care pathways in order to promote shared decision-making and improve the knowledge of the professionals involved. As a secondary objective, the study will provide useful insights for the prevention of decision-making conflicts frequently at the basis of the dispute in the pediatric field.

## Introduction

Medical advances in clinical diagnosis and treatment have revolutionized the natural history of many diseases, favorably changing the prognosis and life expectancy. The achievement of similar results, however, has forced modern medicine to focus on the pathophysiology of diseases and to divert attention from the sick person in its entirety. Consequently, the use of different welfare procedures risks deriving from the uncritical application of protocols rather than from conscious decisions discussed extensively with the patient according to the ethical principles of freedom and responsibility (1).

Despite the considerable progress of medical science over the years, pediatric population, can still be affected by serious illnesses that, lead children and adolescents to experiencing all the clinical, psychological, ethical and spiritual problems related to incurable diseases and death.

In the pediatric field, scientific knowledge has led to the reduction of acute pathological states and shifting the goal of medical care toward chronic conditions, rare and congenital diseases that usually require careful long-term planning and multidisciplinary coordination.

Currently, pediatric patients suffering from chronic and severe diseases are increasingly subjected to invasive diagnostic and therapeutic procedures, mostly related to intensive care. Similar treatments are sometimes applied without a preliminary dialogue aimed at assessing frailty, needs, expected benefits, predictable risks, and quality of life offered. A similar paternalistic relationship between the doctor and the pediatric patient with chronic or serious diseases is understandable in light of the fragility of the individual, but it is to be considered anachronistic in the face of the wide scientific knowledge and care alternatives offered by modern medicine. In this regard, the management of care relationship in such type of patients should be oriented toward respect for autonomy and sharing, in order to prevent controversies and to consolidate the therapeutic alliance.

A growing body of evidence documents the existence of debate among health professionals centered on the opportunity to consider survival as the only primary endpoint, without considering other relevant endpoints such as quality of life after treatment. The generation of adequate answers to the questions discussed is an obligation since the protection of the patient passes through the shared and conscious choice of treatments able to increase survival and improve the prognosis *quoad valetudinem* (2, 3).

Recently, several pediatric care pathways have been created based on the awareness that, especially in the terminal phase of some serious diseases, treatments aimed at the mere prolongation of life are useless or even harmful, and must be replaced by adequate strategies – like palliative care - aimed at respecting the principles of beneficence and non-maleficence. In more detail, it is essential that healthcare professionals adopt the best therapeutic aids and avoid excessive or even harmful treatments (4). Pediatric care needs to be extensively involved in this improvement process (5, 6), precisely because of the importance of avoiding unrealistic assessments of the patient's true needs that, in situations such as emergencies and poor maturation, do not have the time or the tools to express a conscious consent (7). The literature is starting to provide sufficient evidence for issuing recommendations on the behaviors that pediatric healthcare professionals can adopt and on the reference criteria for the evaluation of therapeutic choices, especially in cases where the disease requires a timely choice or use of intensive care.

The approach to children with serious illnesses must consider their biological, psychological, relational, social, and clinical characteristics. These characteristics determine and modulate the type and quantity of the needs presented, conditioning the actions to be taken and the assistance models to be implemented. In fact, in addition to the individual peculiarities and intersubjective variability, serious and incurable diseases cause an increase in the healthcare load precisely due to the complexity of the interaction with the patient and relatives in a phase of life delicate for earliness and health impairment. The great variety of clinical typologies and the variability of situations that can be found in the treatment of pediatric diseases recognize a plurality of causes of considerable interest in understanding the mechanisms of care. The pediatric age includes individuals that are absolutely dissimilar not only for anthropometric variables, but for basic biological structure, metabolism, organ maturity, potential for growth and development (8); undoubtedly the physiology and pathophysiology of a newborn are absolutely unlike those of an adolescent, just as the structure of a baby differs from that of a child. The treatments required are generally complex, with a high degree of care and requiring specific skills and technologies for patients with different biological systems based on age.

The progressive emancipation of the pediatric patient in relation to care decisions is a competitive issue due to the need to protect the rights of information and self-determination considering the different cognitive abilities with respect to adults. Furthermore, particular pediatric medical situations - such as the end of life - are difficult to discuss and more often neglected or inadequately managed, probably due to a gap in communication. The relationship between doctor, patient and caregivers is extremely complex due to the number of parties involved and the sensitivity of the topics covered with respect to the concepts of life and health. Adequate and effective communication presupposes that the healthcare professional interacts with the patient and caregivers in an understandable, firm, empathetic, interested, prompt and reality-related manner. The adoption of a close collaboration between family and doctor is a central element in the management of pediatric patients and should include the child in communication and decision-making processes, in order to guarantee the highest level of well-being possible in respect of his right to self-determination as well as avoiding conflicts and disagreements (9). Consequently it is crucial that the ethics of information, sharing, and consent, are established in the pediatric field as the basis of the care relationship and the decision-making processes (10, 11). Such an implementation of the care process naturally requires a cultural change that centralizes the patient's development and exercise of self-determination skills. In this perspective, therefore, self-determination in the pediatric setting is possible only if health professionals and caregivers direct their efforts to the enhancement of the patient's self-awareness, self-sufficiency, and self-esteem.

The present paper aims to analyze the issues of information quality and sharing in pediatric care pathways in order to promote shared decision-making and improve the knowledge of the professionals involved. As a secondary objective, the study will provide useful insights for the prevention of decision-making conflicts frequently at the basis of the dispute in the pediatric field.

## Shared Decision Making

**Figure 1:**
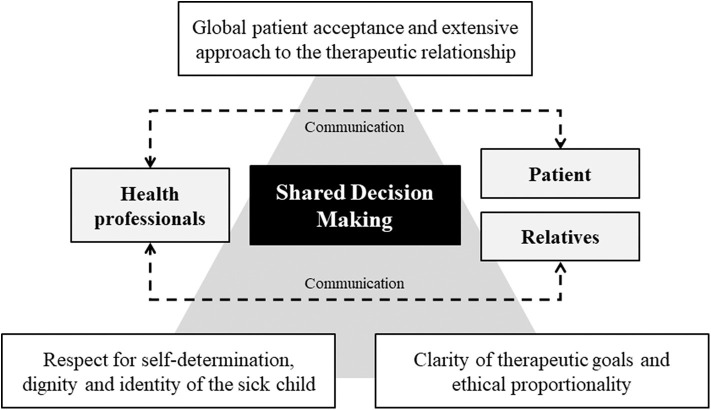
Shared decision making (SDM) process.

The relationship of care centered on the pediatric patient must be aimed at the definition and realization of the primary needs - such as food, accommodation, assistance, appropriate care - compatibly with the limits imposed by the disease. For this reason, it is essential to plan the treatment path based on three principles (Figure 1):

- sharing of decisions in the context of shared and anticipated planning that also involves parents;
- respect for self-determination, dignity and identity of the sick child;
- abstention or interruption of disproportionate treatment.

A cure is “proportionate,” and therefore ethically licit, only if in addition to being clinically appropriate it is consciously accepted by the patient, even pediatric. Obviously, awareness is subordinated to the degree of dependence of the patient, therefore the involvement of the family is essential in sharing therapeutic choices. Clinical appropriateness responds to the reasonable probability that a given treatment, in a given patient, can reach the therapeutic goal by positively modifying the prognosis and providing a reasonable recovery perspective. The acceptance by the patient responds instead to the conscious evaluation of the relationship between benefits and psychophysical burdens that from treatment, even if clinically appropriate, may derive.

Shared decision-making (SDM) can be considered as an extension of informed consent. In particular, it represents a care approach based not only on information and consent, but above all on the close interaction between doctor - holder of scientific knowledge - and patient - owner of inalienable rights - in sharing responsibilities and choices regarding the therapeutic path (12, 13). The sharing process makes it possible to plan the most appropriate assistance for each subject considering the preferences, needs, inclinations, and objectives; nevertheless, a similar implementation of pediatric patient management can determine an increase in adherence to care and satisfaction (14–16).

In the pediatric field, parental participation in the decision-making process is fundamental in order to optimize the understanding of the patient's will without, however, delegating therapeutic choices to the family (17). In consideration of the psychological and emotional impact of pediatric diseases, health professionals are required to protect the dignity of the patient through the support and involvement of family members in the various phases of care (18, 19). Caregivers are instead asked to participate in the decision-making process as facilitators and—where necessary—to decide in accordance with the best interest of the patient. The involvement of the child must be personalized and proportionate to the cognitive-relational possibilities, respecting the centrality of the patient (20–25).

Pediatric departments, especially in the area of emergencies and severe diseases, represent highly specialized and intensive care facilities. Professionals working in similar structures are involved in the treatment of extremely delicate patients due to the need for security and knowledge of the reality that surrounds them. As with other medical branches, the goal of hospitalization in a pediatric ward is to overcome clinical problems in order to promote the recovery of a decent and acceptable quality of life for the sick child.

Sharing decisions in pediatrics constitutes a moment of global acceptance of the patient that is based on the control of pathological manifestations, attention to the human and social aspects of the disease, in the relationship with family members, in psychological and spiritual support. The effectiveness of the sharing and the good quality of the care relationship can be defined based on the clarity of the therapeutic objectives, the awareness and acceptance by the patient and the parents, the ethical sustainability and the agreed definition of the limits between intensive and palliative care (26, 27).

The extensive approach to the therapeutic relationship makes it possible to achieve fundamental goals of the healing process such as understanding the views, sensitivity, basic needs and expectations of the patient and their families. Finally, this kind of involvement allows the decisions to be calibrated in full compliance with the values of all parties involved in the care process (28).

According to several ethical and legal orientations, the time spent in communication is in effect a part of the therapeutic process. The cornerstones of good communication between the pediatricians, the patients and their families can be summarized as follows:

- correct and understandable description of the patient's condition;
- consistency and homogeneity of the information provided by the different healthcare professionals involved in the care path;
- gradualness of the information process;
- attention to the need for information at all times, in order to avoid potential negative repercussions (compliance, despair, etc.) on the sick child and the family;
- two-way information flow;
- empathy and ability to induce the patient to externalize his own emotion;
- ability to prevent possible conflicts with family members and family members by constantly checking the degree of understanding of the information provided;
- availability to dialogue with preparation of the necessary means.

Communication is not only information, but it is mainly an interaction in which the transmission of the message content is a mere element. Clear, transparent and comprehensive communication is undoubtedly the best tool for optimizing the care path and conflict prevention. Communication and therapeutic relationship are inextricably correlated. In fact, the quality of communication is directly proportional to the trust and reciprocity of listening in the relationship between doctor and patient; in the same way, a good relationship is fed by communication conceived as an integral part of the care process.

## Advance Care Planning

**Figure 2:**
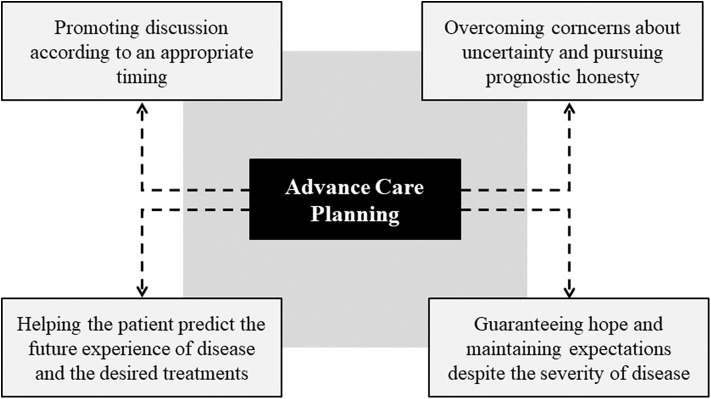
Advanced care planning (ACP) process.

Advanced Care Planning (ACP) is an important planning tool that allows to share in advance the most important therapeutic choices in the case of life-limiting or life-threatening conditions (Figure 2). Despite representing an extremely relevant tool, ACP constitutes a particularly bumpy route in the pediatric field due to medical and ethical reasons, as well as to the contingent communication and decision-making difficulties. At first, the duty of timely communication is conditioned by uncertainty since the prognosis in the pediatric patient can be particularly challenging and inaccurate, also considering the heterogeneity of diseases (29–31). On the other hand, it is evident that the development of communication in the prognostic field is important for the development of news, for the possibility of making considered and informed choices, as well as for improving the quality of life of the patient (32).

The discussion on care provisions should focus on the multiple facets of the possible therapeutic approaches, with the assessment of curative and palliative care, management strategies for emergency situations and patient's will on resuscitation. The determination of the child's goals and expectations is fundamental for psychological well-being and the care relationship, as it allows to address multiple aspects of end-of-life assistance including home care (33).

The ACP process protects the patient's autonomy and improves end-of-life healthcare. Consistent with several scientific contributions, the benefits deriving from an adequate Advance Care Planning consist in the reduction of aggressive treatments, the improvement of the quality of life in its terminal phases, the reduction of hospitalizations, and the greater adherence of care to the patients' needs (34–37). Nevertheless, the supporting function of the ACP in the elaboration of severe diseases and in the preparation of pediatric patients and relatives to the terminal phase of life is ascertained (38).

In opposition to the relevant positive implications, the ACP in the pediatric field presents some barriers mainly related to the young age of the patient and consequently to the difficulties of involvement in discussions concerning the treatments to be implemented in case of deterioration of health conditions (39, 40). Similarly, the ACP process can be limited by cultural and emotional factors regarding the possibility for the sick child and family members to foresee the future experience of illness and, therefore, the desired treatments (41). On the other hand, barriers that hinder the activity of health professionals can be identified; in particular, there is a widespread concern among pediatricians that prognostic honesty may cause an unjust harm or even a destruction of the patient's expectations (42). Similar concerns are understandable given the specific weight that characterizes hope in the pediatric context.

Despite the described barriers, Advance Care Planning should always be encouraged and performed with an appropriate timing even if patients and families seem unprepared. Indeed, not infrequently patients and relatives are unaware of the need for the ACP or are waiting for the health professional to introduce the discussion (43). Dialogue with the sick child and family members offers the possibility of guaranteeing hope and maintaining expectations despite the severity of the prognosis (44). In light of the above, it is clear that the perception of barriers and the lack of dialogue on the ACP constitute a knowledge gap for health professionals.

## Good Practice Statement

Conclusively, different issues concerning the modalities of pediatric assistance in severe or progressive diseases require a deepening by the scientific community. The correct management of pediatric assistance implies a complex relationship between healthcare professionals, patient and family members, but above all a particular sensitivity of health structures and adequate training of the personnel involved.

The present study has highlighted the lack of organization of pediatric care pathways in certain contexts, in particular as regards precisely the processes of information, consent and sharing as well as the protection of self-determination.

In order to prevent the occurrence of prejudices and implement care pathways in such delicate areas as severe diseases and end of life it is necessary to propose recommendations of good pediatric practice.

In the first place, it seems advisable to support the dialogue through the identification of communicative strategies useful for the management of uncertainty characterizing some clinical conditions; through a communicative process of good quality it is in fact possible to limit uncertainty and guide conscious choices.

Secondly, despite the concerns related to the effects of the communication of bad news on the patient and family members, prognostic honesty must be considered fundamental. The transparent sharing of all the implications of the disease represents an opportunity for the protagonists of the care path to prolong the treatment time and jointly formulate hypotheses about the future.

It is also recommended to consider communication, understanding, informed consent, therapeutic choices and prognoses not only as procedural elements but also as aspects underlying the subjective expressions and around which the care relationship is built. The availability, serenity and clarity of the dialogic exchange, as well as the more frequent use of explicit contents, facilitate the possibility of managing emotional experiences more easily, of integrating subjective experiences and of adequately involving the pediatric patient in the sharing process.

Finally, it is advisable to direct the different phases of the care pathway toward strengthening the autonomy and centralizing emotions in order to improve the comprehension skills of the interlocutors, improve the communication process and promote the sharing of therapeutic choices.

## Author Contributions

All authors contributed to the drafting and critical revision of the work. All authors have approved the final version of this manuscript.

## Conflict of Interest

The authors declare that the research was conducted in the absence of any commercial or financial relationships that could be construed as a potential conflict of interest.
